# Supplementary material for: A BRRF1-CCR4-NOT axis underlies conserved transcriptome-wide loss of splicing fidelity during gammaherpesvirus reactivation
Source: bioRxiv. 2026 May 23:2026.05.20.726682. Preprint. [Version 1] doi: 10.64898/2026.05.20.726682 (PMC13228557; doi:10.64898/2026.05.20.726682)
Supplement: 7 [file NIHPP2026.05.20.726682v1-supplement-7.pdf]

bioRxiv preprint doi: https://doi.org/10.64898/2026.05.20.726682; this version posted May 23, 2026. The copyright holder for this preprint (which was not certified by peer review) is the author/funder, who has granted bioRxiv a license to display the preprint in perpetuity. It is made available under a CC-BY 4.0 International license.

Supplemental Figure Legends

Supplemental Figure S1. Differential alternative splicing analyses of EBV and KSHV reactivations
The number of differential AS events in KSHV reactivation model (FDR < 0.0005) is shown as a bar plot. The percentage of statistically significant events (Neg-IncDiff and Pos-IncDiff) for each AS type relative to the total number of significant events across all five AS types is shown as pie charts for EBV and KSHV reactivation models (FDR < 0.0005).

Supplemental Figure S2. Antigenicity analysis of reactivation-derived neopeptides
The neopeptides shared among our EBV+ B cell, and B and epithelial cell reactivation models were analyzed for their predicted antigenicity using IAPred (38). (**A**) The number of peptides that were predicted to have high (> 0.3), low (< -0.3), or moderate (-0.3 to 0.3) for B cell and B and epithelial cell reactivation models. (**B)** The number of neopeptides predicted to have high antigenicity shared among B and among B and epithelial cell reactivation models. (**C**) Density distribution of intrinsic antigenicity scores for neopeptides shared among B cell reactivation models (blue) and shared B and epithelial cell reactivation models (orange). (**D**) Correlation between intrinsic antigenicity score and neopeptide length for high-scoring neopeptides (> 0.3). Each point represents a neopeptide shared among B cell reactivation models (blue) and among B and epithelial cell reactivation models (orange). Top 15 selected genes corresponding to the highest antigenicity scores are indicated.

Supplemental Figure S3. Tissue enrichment analysis of genes corresponding to highly antigenic neopeptides
Genes corresponding to predicted highly antigenic neopeptides were analyzed for tissue enrichment using STRING (39). Enriched gene sets were considered statistically significant at FDR < 0.05.

Supplemental Figure S4. Significant exon 2 skipping in CDK2
(A) A 3D structural model of the CDK2-CCNE1 complex was predicted using AlphaFold3 and visualized in PyMOL. Exon 2 in CDK2 (magenta) and exon 8 in CCNE1 (red) are indicated. In addition to the skipped exon regions, other regions in both CDK2 and CCNE1 appear to contribute to the interaction interface. This model was used to identify interface residues involved in the CDK2-CCNE1 interaction. **(B)** The fraction of exon 2 skipping in CDK2 was calculated using the same approach as for CCNE1 and TYK2 in **Figure 4**.

Supplemental Figure S5. BRRF1 exhibits splicing-disrupting phenotype
(**A**) Workflow of systemic analysis of the effect of each EBV early gene expression on cell splicing changes. The EBV-negative Burkitt lymphoma cell line DG75 was co-transfected with CMVp-GFP and a pLVX plasmid expressing either a control or each EBV early gene open reading frame (ORF). After 24 hours, GFP+ cells were selected from each sample by FACS (N=3 or 4 per group). The panel was created using BioRender.com. (**B**) Fraction of expressed genes exhibiting increased exon skipping in EBV reactivation models, the top five EBV early genes with the most significant influence on splicing changes, and knockdowns of 186 RNA-binding proteins (RBPs) using SEFractionExpressed module (SpliceTools; FDR < 0.0005; TPM ≥ 3 in either condition). (**C**) Fraction of increased SE events that are unannotated in EBV reactivation models and in the top five EBV early genes with the greatest impact on increased exon skipping, quantified using SEUnannotated module (SpliceTools; FDR < 0.0005).

Supplemental Figure S6. Knockdown of CCR4-NOT subunits
(**A**) Workflow of CCR4-NOT subunit knockdowns in DG75 (*left panel*) and pCEP4-BMRF1p-GFP Mutu cells (*right panel*)**.** (*left panel*) DG75 cells were transfected with either mock siRNAs (siCntl) or CNOT-specific siRNAs (siCNOT**)** for 48 hours (CNOT1), 6 hours (CNOT6), or 18 hours (CNOT9). The samples were then split into two groups, one that was co-transfected with the CMVp-GFP plasmid along with either the BRRF1 expression plasmid or the control (pLVX) plasmid. This produced four experimental conditions: siCntl-pLVX, siCntl-BRRF1, siCNOT-pLVX, and siCNOT-BRRF1. After 24 hours, GFP+ cells were sorted using FACS. (*right panel*) A similar experimental setup was conducted in pCEP4-BMRF1p-GFP Mutu cells. After 24 hours of the first transfection with either mock or CNOT1-specific siRNAs, the samples were split into two groups. One group was reactivated with SV40p-Zta, while the other group was transfected with SV40p-Cntl (SVpE) for 24 hours. This setup resulted in four experimental conditions as shown in the figure. GFP+ and GFP-cells were sorted using FACS. The panel was created with Biorender.com. (**B-E**) RT-qPCR analysis for knockdown of

bioRxiv preprint doi: https://doi.org/10.64898/2026.05.20.726682; this version posted May 23, 2026. The copyright holder for this preprint (which was not certified by peer review) is the author/funder, who has granted bioRxiv a license to display the preprint in perpetuity. It is made available under a CC-BY 4.0 International license.

CCR4-NOT subunits. CNOT1 (**B**)**,** CNOT6 (**C**)**,** and CNOT9 (**D**) knockdown in DG75 and CNOT1 knockdown in pCEP4-BMRF1p-GFP Mutu (**E**).

Supplemental Figure S7. **Sequence and structural homology between BRRF1 and KSHV ORF49**
(**A**) Protein sequences of EBV BRRF1 and its homolog in KSHV, ORF49, were aligned using Clustal Omega (36). Identical residues are indicated by (*). The percentage sequence identity between BRRF1 and ORF49 was calculated using BLASTp suite (NCBI) (37). (**B**) The ORF49-CNOT9 interaction interface was modeled using AlphaFold3. The CIYE motif (Cysteine-Isoleucine-Tyrosine-Glutamic acid) at the position 224-227, which is highly conserved with the CIYY motif in BRRF1, is predicted to contribute to CNOT9 binding. Similarly to BRRF1, tyrosine 226 in ORF49 is predicted to be a critical residue mediating interaction with CNOT9**.** (**C**) The expression level of BRRF1 mutants. From the co-IP experiment performed to validate the interaction between BRRF1 mutants and CNOT9, anti-FLAG antibody was used to assess the expression levels of BRRF1 mutants relative to BRRF1-WT. (**D**) Structural homology analysis of BRRF1-WT and mutants. Structure of BRRF1-WT and BRRF1 mutants was predicted individually using Alphafold3. Each mutant structure was aligned to BRRF-WT in PyMOL, which gave root-mean-square deviation (RMSD) values to assess structural similarity. An RMSD < 2Å indicates high structural similarity.

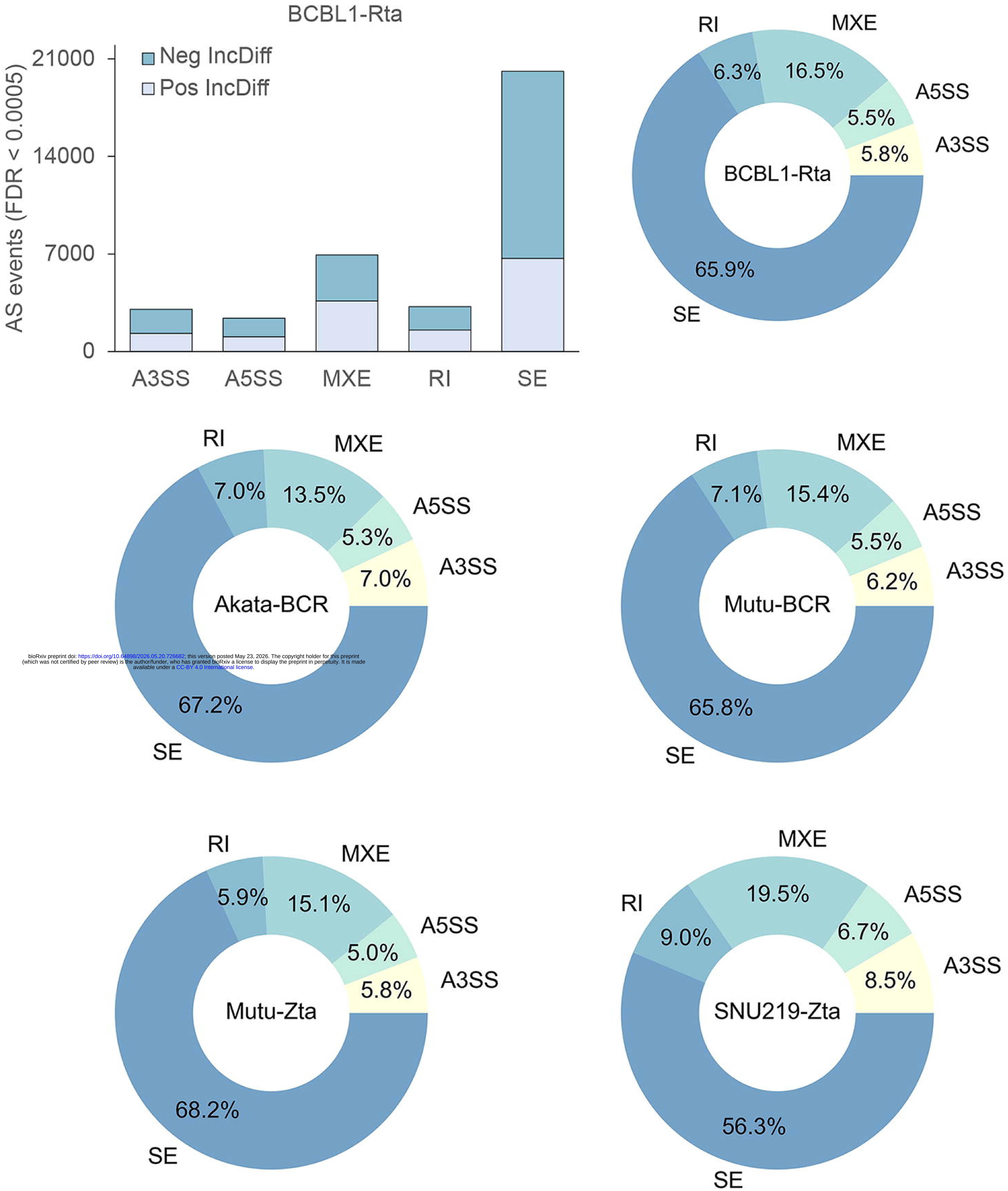

bioRxiv preprint doi: https://doi.org/10.64898/2026.05.20.726682; this version posted May 23, 2026. The copyright holder for this preprint (which was not certified by peer review) is the author/funder, who has granted bioRxiv a license to display the preprint in perpetuity. It is made available under a CC-BY 4.0 International license.

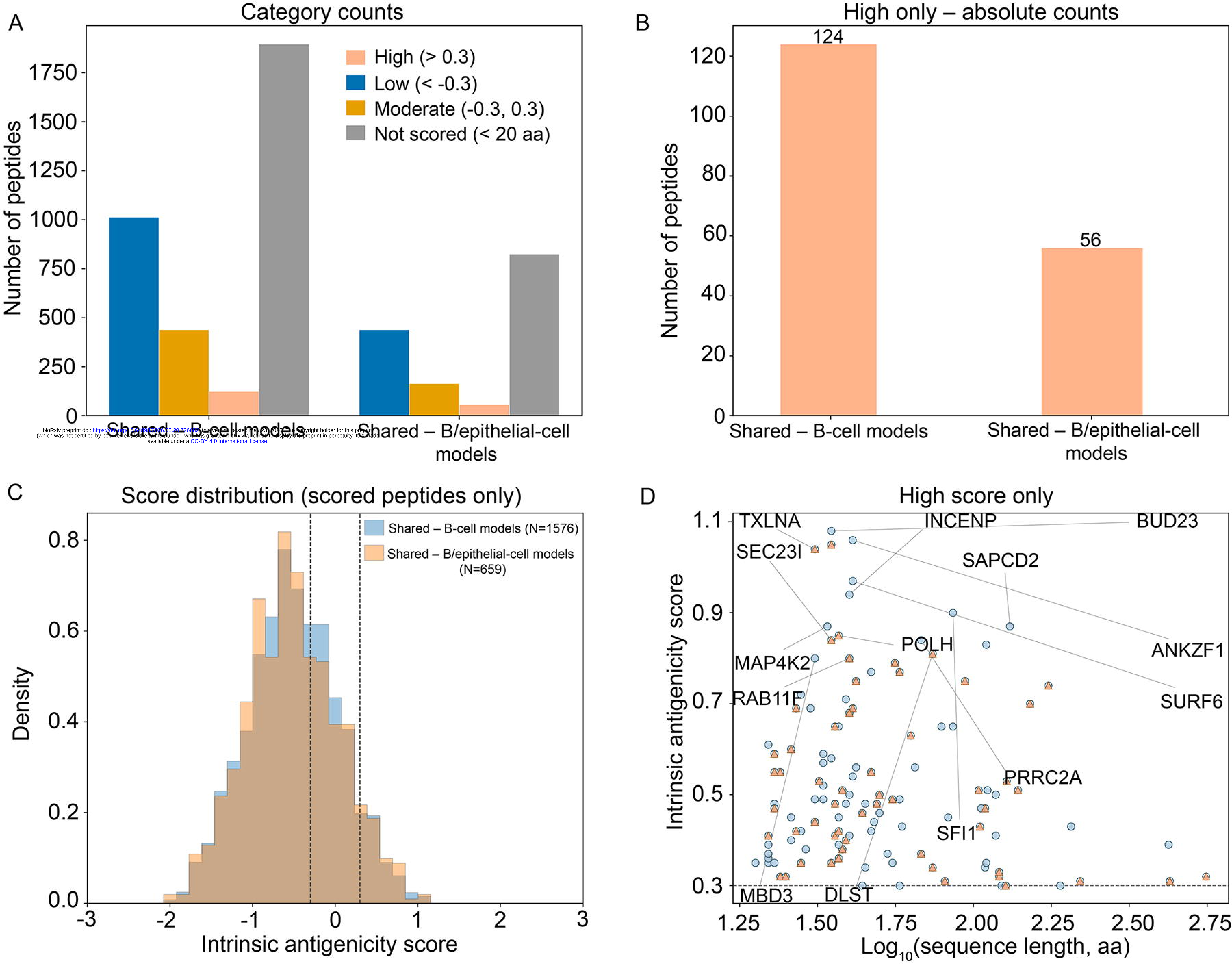

bioRxiv preprint doi: https://doi.org/10.64898/2026.05.20.726682; this version posted May 23, 2026. The copyright holder for this preprint (which was not certified by peer review) is the author/funder, who has granted bioRxiv a license to display the preprint in perpetuity. It is made available under a CC-BY 4.0 International license.

bioRxiv preprint doi: https://doi.org/10.64898/2026.05.20.726682; this version posted May 23, 2026. The copyright holder for this preprint (which was not certified by peer review) is the author/funder, who has granted bioRxiv a license to display the preprint in perpetuity. It is made available under a CC-BY 4.0 International license.

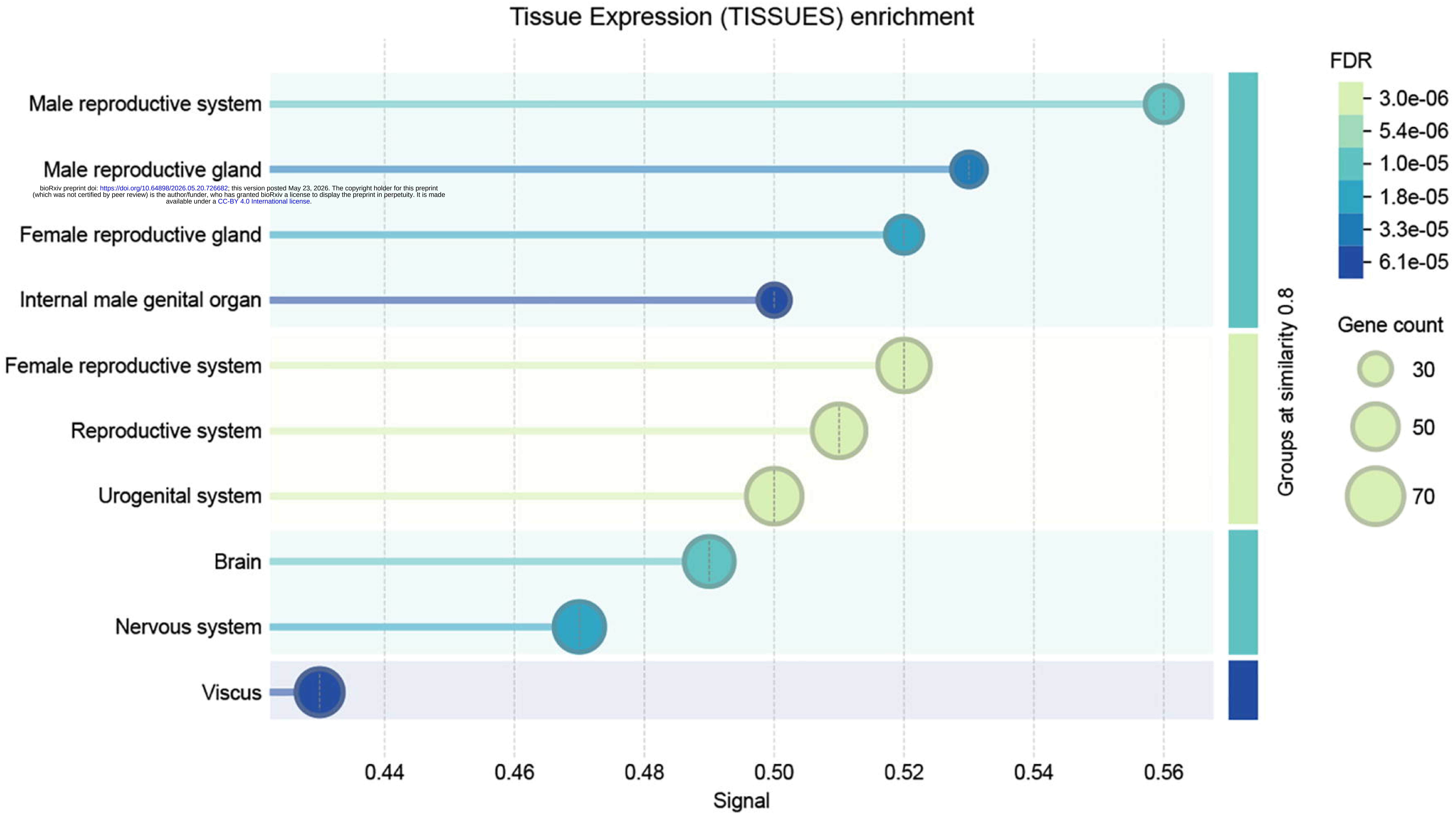

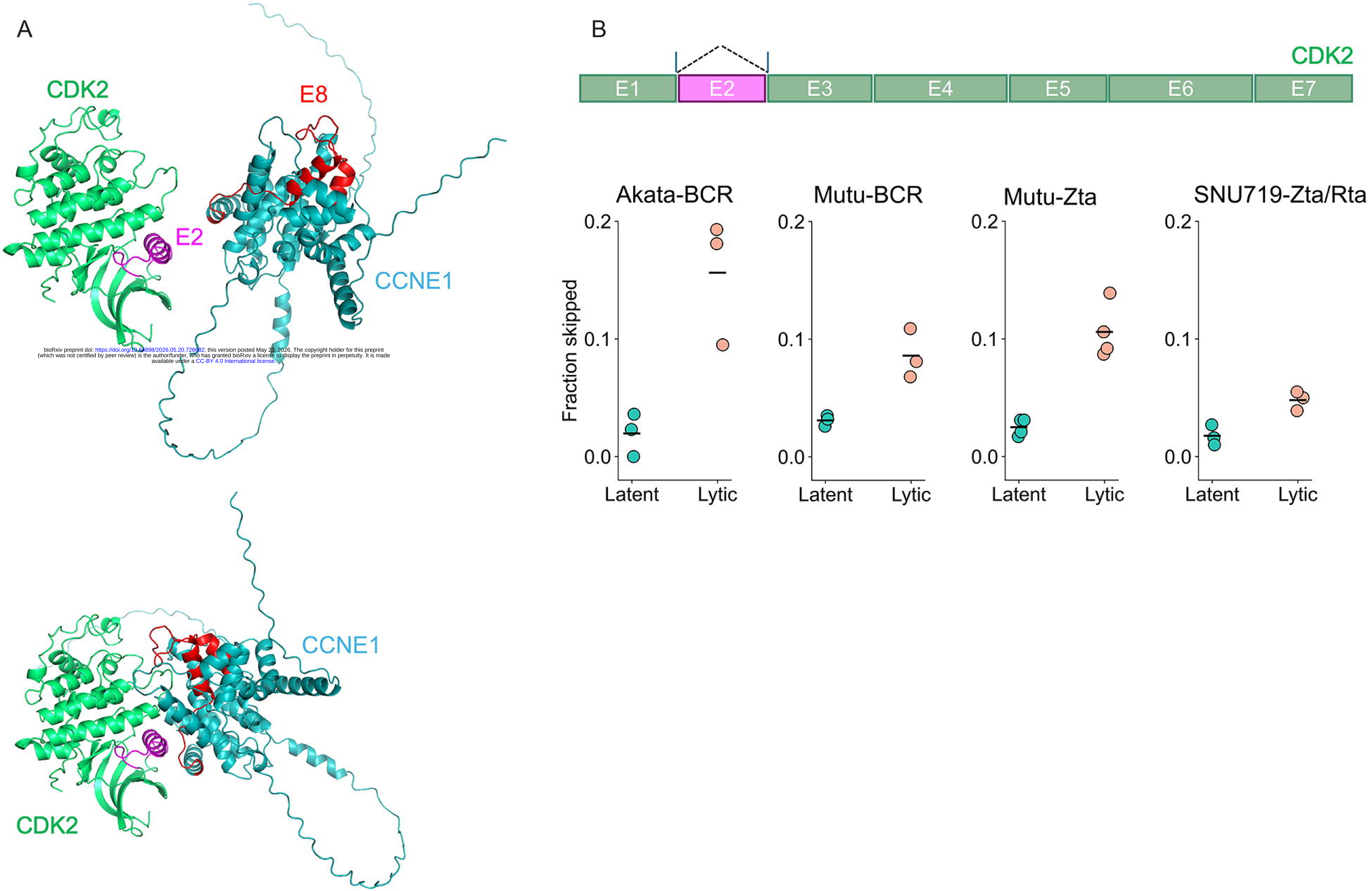

bioRxiv preprint doi: https://doi.org/10.64898/2026.05.20.726682; this version posted May 23, 2026. The copyright holder for this preprint (which was not certified by peer review) is the author/funder, who has granted bioRxiv a license to display the preprint in perpetuity. It is made available under a CC-BY 4.0 International license.

bioRxiv preprint doi: https://doi.org/10.64898/2026.05.20.726682; this version posted May 23, 2026. The copyright holder for this preprint (which was not certified by peer review) is the author/funder, who has granted bioRxiv a license to display the preprint in perpetuity. It is made available under a CC-BY 4.0 International license.

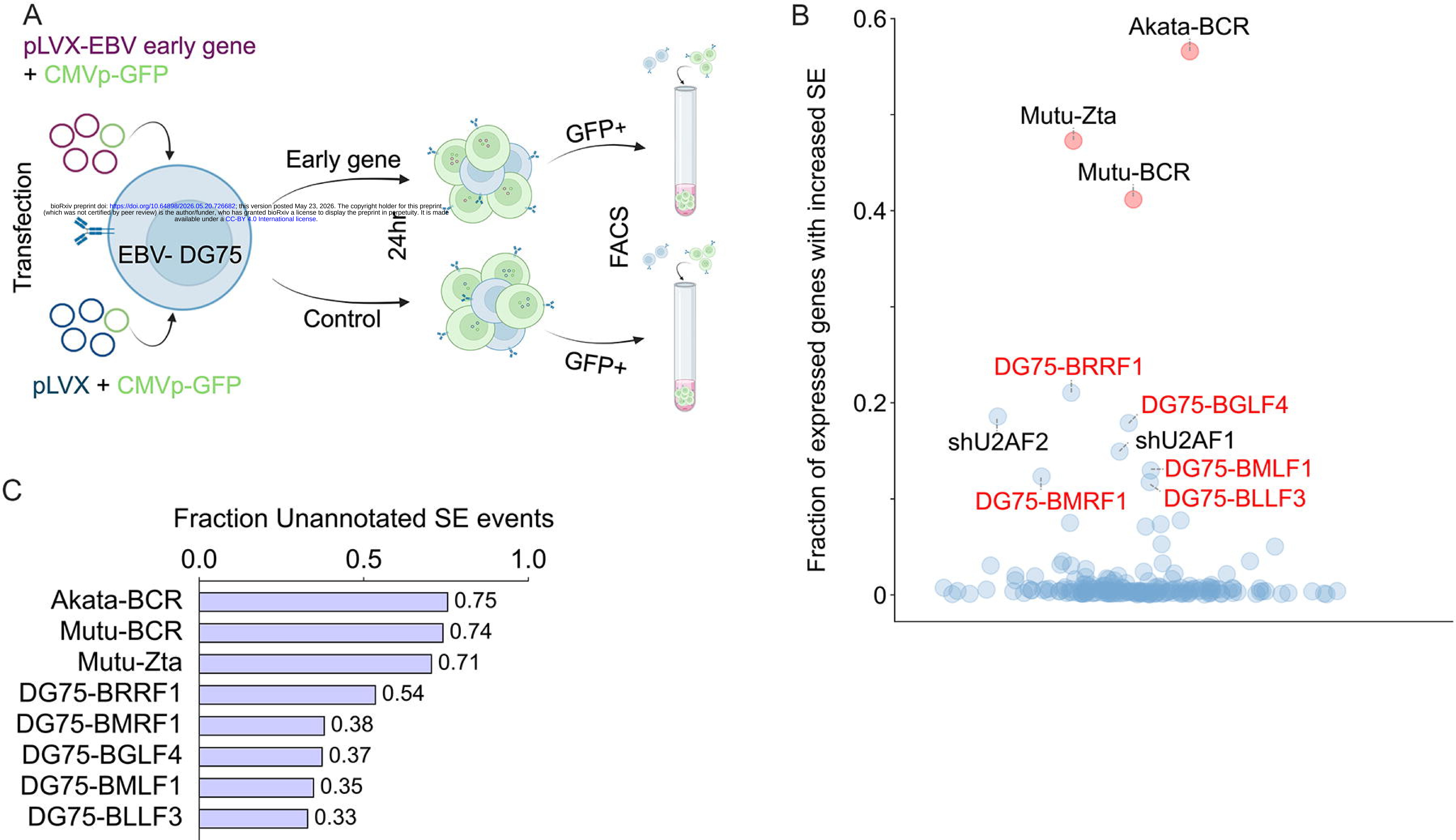

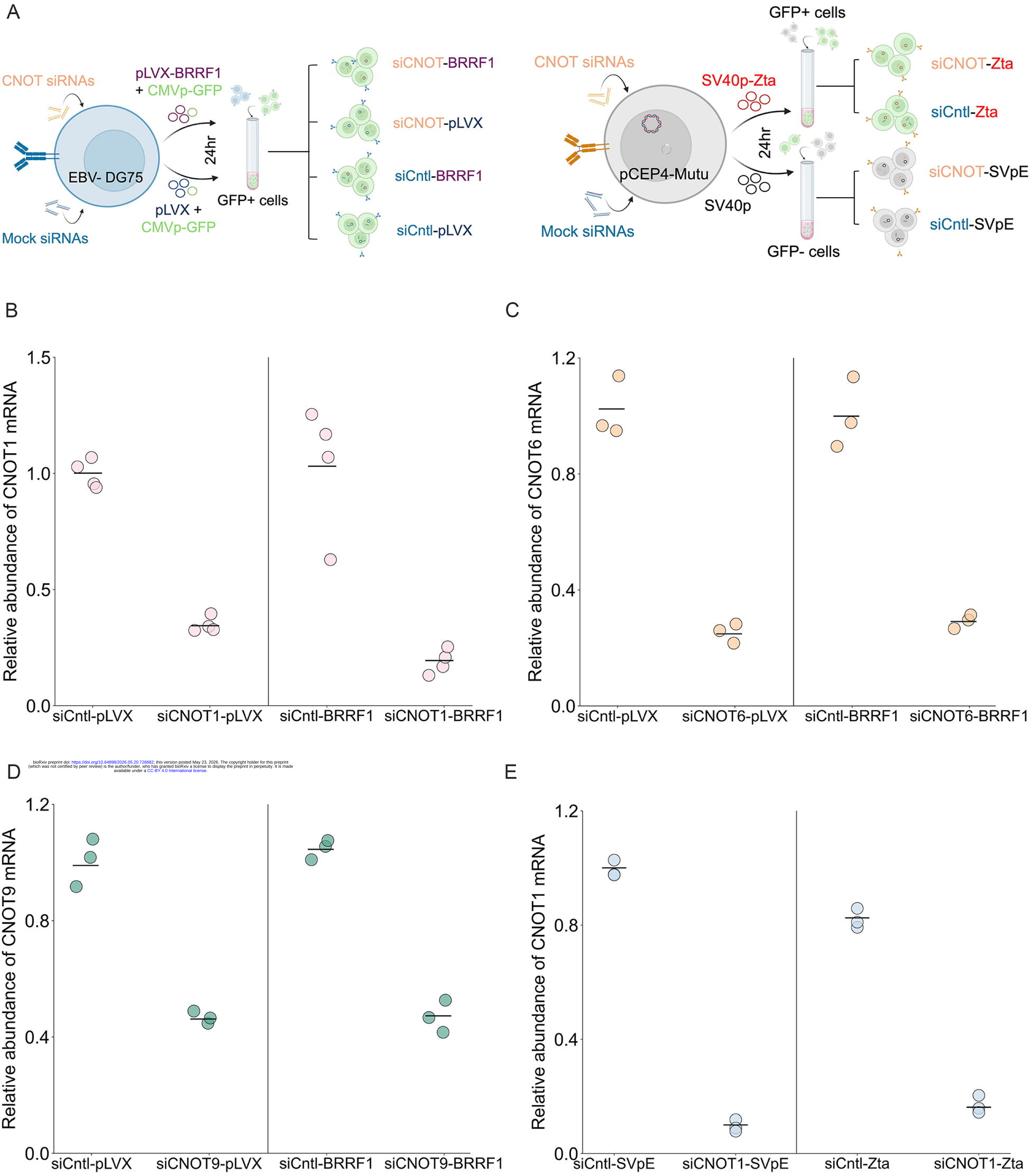

bioRxiv preprint doi: https://doi.org/10.64898/2026.05.20.726682; this version posted May 23, 2026. The copyright holder for this preprint (which was not certified by peer review) is the author/funder, who has granted bioRxiv a license to display the preprint in perpetuity. It is made available under a CC-BY 4.0 International license.

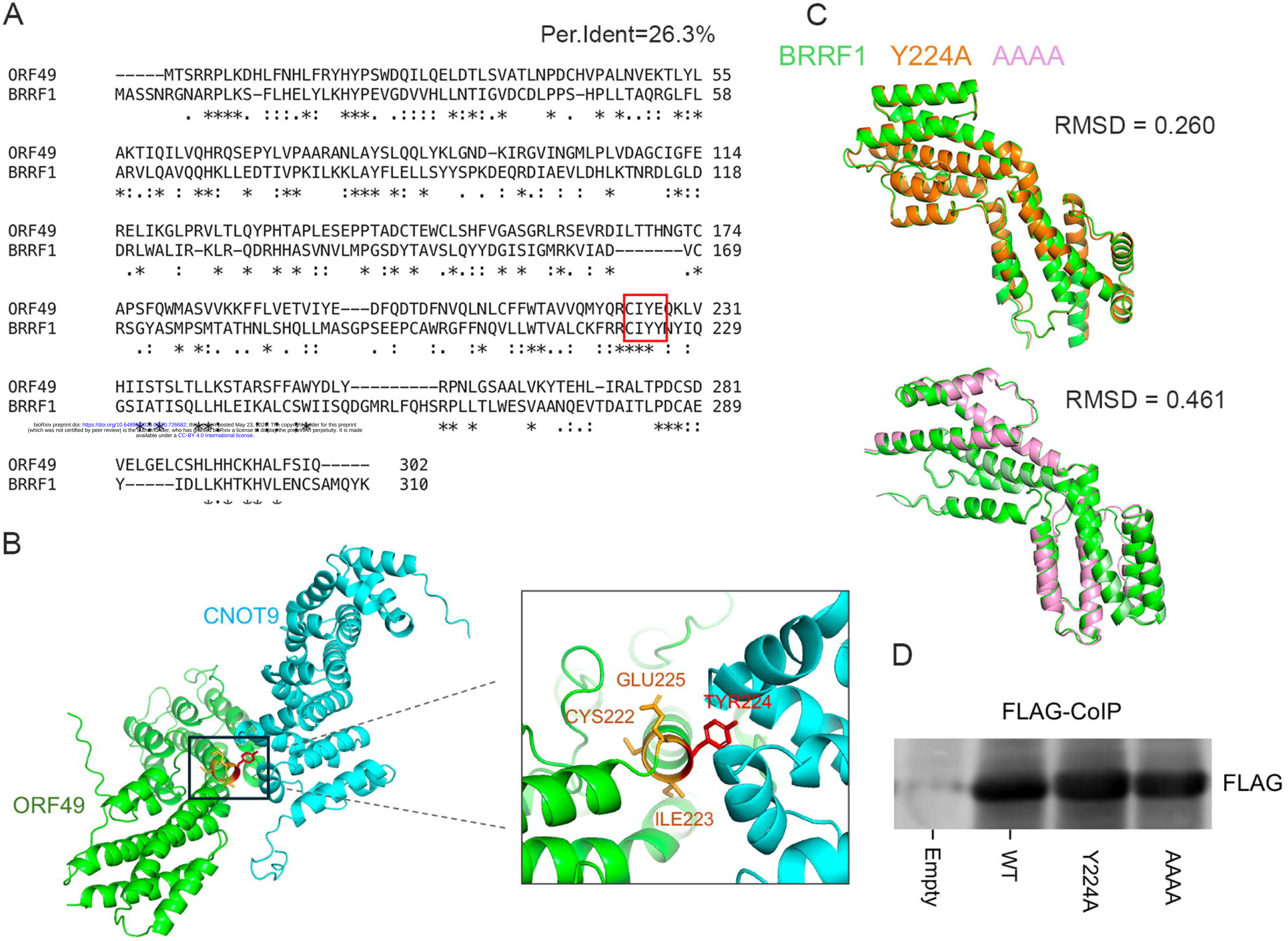

bioRxiv preprint doi: https://doi.org/10.64898/2026.05.20.726682; this version posted May 23, 2026. The copyright holder for this preprint (which was not certified by peer review) is the author/funder, who has granted bioRxiv a license to display the preprint in perpetuity. It is made available under a CC-BY 4.0 International license.
